# Male medaka continue to mate with females despite sperm depletion

**DOI:** 10.1098/rsos.241668

**Published:** 2025-01-08

**Authors:** Yuki Kondo, Masanori Kohda, Satoshi Awata

**Affiliations:** ^1^ Laboratory of Animal Sociology, Department of Biology, Graduate School of Science, Osaka City University, Osaka 558-8585, Japan; ^2^ Laboratory of Animal Sociology, Department of Biology, Graduate School of Science, Osaka Metropolitan University, Osaka 558-8585, Japan

**Keywords:** sperm depletion, fertilization rate, mating behaviour, medaka, *Oryzias latipes*

## Abstract

In animals where males engage in multiple matings, sperm depletion can substantially reduce the reproductive success of both sexes. However, little is known about how successive matings affect sperm depletion, fertilization rates and mating behaviour. Here, we investigated this phenomenon under laboratory conditions. Medaka (*Oryzias latipes*), an externally fertilizing fish, is an ideal model to test predictions of sperm depletion because there are established methods to observe its mating and count sperm. Medaka males mated with multiple females (19 per day, on average; range, 4–27), experiencing significant sperm depletion, with sperm release declining markedly after the first few matings, reaching only 0.5–6.3% by the last mating of the day. Fertilization rates decreased, particularly after approximately 10 consecutive matings, although there was some recovery in the next-day’s matings. The decline in courtship effort and mating duration probably resulted from the males becoming increasingly fatigued. Despite the reduced sperm availability, females did not adjust their clutch size as a counterstrategy. These results suggest substantial reproductive costs for males and the potential for sexual conflict owing to limited sperm availability. For species with frequent successive mating, these findings highlight the need to reconsider reproductive strategies and their impact on sexual selection.

## 1. Introduction

The energy cost of sperm production is far from trivial, and sperm and ejaculation are now widely perceived as costly for males [1–5]. Under these circumstances, sperm allocation theory predicts that males should strategically allocate available sperm to maximize their fitness [6,7]. This prediction is supported empirically; males of many animals, such as insects, birds and mammals, adjust their sperm investment according to the risk and intensity of sperm competition, or in response to female quality [8]. Strategic sperm allocation is more pronounced in serial than in single mating, particularly when males are susceptible to sperm depletion [9–12]. This is because sperm depletion, in which a male’s stock of sperm is reduced or exhausted, can reduce the ability of males to fertilize eggs during subsequent mating events. Thus, sperm depletion can considerably affect male fitness, restricting the potential to capitalize on additional mating opportunities and reducing the benefits of multiple matings.

Theoretical studies have demonstrated that males struggle to maintain elevated mating rates, which results in a progressive reduction in ejaculate size [1,9]. Indeed, males may suffer from sperm depletion during successive matings, with fewer sperm released in later matings than in the first mating [9,13–20]. Nevertheless, the limitations on male reproductive capacity during successive matings have rarely been examined empirically. For instance, although *Drosophila* mating behaviour has been studied for decades, surprisingly little is known about the number of times a male can mate on any given time-scale (but see Douglas *et al*. [18]). The relative dearth of research on this topic may stem from the implicit assumption that any upper limits on male mating capacity are so high as not to impede male reproductive success. By contrast, male mating strategies during the first mating have been studied extensively. Consequently, little is known about the potential upper limits of male mating frequency, and the associated ejaculation and reproductive strategies of the sexes. Ignoring the limitations of male mating frequency and potential reproductive rate (the number of offspring produced per unit time that each sex achieves when mate availability is unlimited) [21] can lead to incorrect predictions about reproductive behaviour and erroneous conclusions about the mating systems of the species.

To determine whether sperm depletion limits reproductive success, it is necessary to assess the released sperm count and changes in fertilization success, number of spawned eggs and male and female behaviours during successive matings. However, in externally fertilizing animals, such as fishes, ascertaining the precise ejaculate and clutch size is challenging owing to the immediate dispersion of gametes in the water [22]. Consequently, simultaneous changes in fish ejaculate size and fertilization rates have been observed in only a few studies. For instance, in a study on the lemon tetra *Hyphessobrycon pulchripinnis*, fertilization rates declined as the frequency of male mating increased, suggesting that males experienced sperm depletion (sperm were not counted on release) [23]. Conversely, in the stickleback *Gasterosteus aculeatus* and bitterling *Rhodeus amarus*, although fertilization rates were not measured, the released sperm count declined as mating frequency increased, suggesting that males suffer sperm depletion [24,25]. In sticklebacks, males that had mated more than once ejaculated approximately 92% fewer sperm per mate than those that mated for the first time. In bitterlings, the released sperm count per mating was 70% lower during the second mating than during the first.

Sperm depletion affects the reproductive success of both males and females. In insects and crustaceans, females that mate with sperm-depleted males are unable to receive sufficient sperm to fertilize all their eggs [26,27]. Furthermore, female insects that mate with sexually experienced males exhibit lower lifetime reproductive success than those that mate with virgin males owing to the reduced ejaculate size and sperm quality of the former males [28,29]. Consequently, male mating history greatly affects female fertility, and females may incur risks by mating with males that potentially suffer from sperm depletion after consecutive matings. Therefore, females experiencing sperm depletion may employ counterstrategies. Nonetheless, knowledge of female reproductive potential and counterstrategies remains limited. In two studies of female counterstrategies, females were able to recognize male mating status and preferred males with less mating experience [30,31]. In the moth *Lobesia botrana*, the number of sperm transferred by virgin males was five times greater than that transferred by non-virgin males, and mating with non-virgin males significantly reduced female fertility. Consequently, females are more inclined to remate after mating with non-virgin males [10]. Considering these findings, it is crucial to investigate female and male reproductive strategies to understand the effects of sperm depletion on reproductive success.

Medaka (*Oryzias latipes*), an externally fertilizing fish, is ideal for testing predictions regarding sperm depletion and the associated behavioural changes, as relevant mating observation protocols and sperm-counting methods have been established [32,33]. Medaka, a small freshwater fish (standard length, 20−30 mm), is native to marshlands and rice paddy channels in southern Japan, where the reproductive season extends from April to September [34,35]. Under laboratory conditions, both sexes mate almost daily in the morning; however, their spawning frequencies in the wild remain unknown [36]. Females spawn once per day, whereas males ejaculate multiple times per day [35,37]. Spawning typically occurs in pairs in the morning, and the spawning behaviour has been documented in detail [32,33,38,39]. Males increase their released sperm count when mating with larger females, or when the risk of sperm competition is higher [32,33]. For individual males, fertilization rates decrease with successive matings; therefore, sperm depletion may occur. Courtship rates may serve as honest indicators of male fertilization capability, and females may respond to male sperm depletion by reducing their clutch size [37]. However, the extent to which the released sperm count changes with successive mating remains unclear. Moreover, the number of eggs remaining in the ovaries has not been investigated by dissecting postspawning females [36]. Therefore, it remains to be clarified whether females genuinely reduce their clutch size in response to successive mating.

Here, we investigated sperm depletion and its effects on fertilization rates, courtship and mating behaviours during successive mating. We allowed medaka males to mate with multiple females consecutively within 1 day until the males stopped mating. We examined the number of sperm and eggs spawned and the fertilization rate and observed male and female behaviours. The eggs remaining in the ovaries after spawning were counted following dissection. We aimed to test three predictions derived from the sperm depletion hypothesis: (i) male fertilization ability declines over successive matings, owing to sperm depletion; (ii) with successive matings, male courtship behaviour decreases, mating latency (the period between meeting a female and starting mating) increases and mating duration declines; and (iii) females reduce their clutch size as a counterstrategy when mating with sperm-depleted males.

## 2. Material and methods

### 2.1. Maintenance

We obtained medaka (himedaka) from a local pet shop and maintained them under the following rearing conditions for one month. Five male and five female medaka were kept in 27 holding tanks (21 × 25 × 35 cm) at the laboratory of Osaka City University (Osaka Metropolitan University), Osaka, Japan. The tanks were illuminated using standard fluorescent lamps for 14 h per day (8.00−22.00) and water temperature was maintained at 26−28°C. The fish were fed Tetra fish food (Tetra, Melle, Germany) three times a day. Males and females were allowed to mate freely in the holding tanks.

### 2.2. Experimental methods

The experiments were conducted from August to September 2021. The experimental protocol was as described previously [32,33]. On the day before the experiment, between 18.00 and 19.00, randomly selected males and females from different breeding tanks were placed separately in small glass tanks (13.0 × 8.0 × 15.0 cm) with opaque partitions to prevent them from interacting. The mating experiments began at 8.00 on the following day. All experiments were conducted using 500 ml of water in each tank (15.8 × 13.0 × 17.0 cm; outer length × outer width × outer depth; glass thickness: 0.3 cm). Water depth, 2.7 cm, did not influence mating behaviour, as medaka typically mate at comparable depths in the wild [33] (Y. Kondo, 2020 personal observation).

A male and female were introduced into an experimental tank, and their behaviour was recorded from the two sides (front and bottom) of the tank using an HDR-CX485 video recorder (Sony, Tokyo, Japan) until mating was complete. The fish were observed until the end of mating or for 20 min if mating did not occur. The male was then removed from the tank and placed in another experimental tank containing a new female. This process was repeated until the male failed to mate with the three consecutive females.

After mating, females carried their eggs beneath their abdomens. The eggs were gently removed from the females and transferred to Petri dishes to assess fertilization rates. One hour after mating, when dividing cells were discernible within fertilized eggs, fertilized and unfertilized eggs were counted under a light microscope (Nikon SMZ−1B; Tokyo, Japan). Females that remained unmated during the 20 min trial were introduced into a tank containing two males (20.0 × 20.0 × 20.0 cm). The fact that all these females mated within 1 h verifies that all females were ready to mate at the start of the experiment. Following anaesthesia with a 1 : 2000 dilution of FA100 (DS Pharma Animal Health, Osaka, Japan), as per the manufacturer’s guidelines [33,40], fish body mass was measured to the nearest 0.01 g. Using photographs and ImageJ 1.50i (NIH, Bethesda, MD), the standard length was determined to the nearest 0.01 mm. The fish were fed before and immediately after the experiments.

To count the remaining eggs in the ovaries, the females of the first and last two matings were sacrificed via an overdose of anaesthetic, fixed in 10% formalin for 1 day, and dissected. The remaining females were returned to the holding tanks. Once individuals were used in the experiment, they were not used again.

To determine whether the males were able to mate the day after the experiment, they were placed in a 13.0 × 8.0 × 15.0 cm glass tank and kept in isolation until the next day. Sexually mature females as mating partners were transferred from the holding tanks to 13.0 × 8.0 × 15.0 cm glass tanks with opaque partitions between 18.00 and 19.00. The next morning, from 8.00, the mating behaviour was observed in the same manner as in the mating experiment. If mating did not occur within 20 min, a different female was presented, and the mating behaviour was monitored again. Once mating occurred or after three changes in the female, the experiment was terminated.

### 2.3. Released sperm count

A previously described procedure [32,33] was used to count the number of sperm released into the tank. Briefly, immediately after each mating, 500 ml water in the tank containing the sperm was vigorously mixed with a glass rod in a 600 ml glass bottle. For sperm fixation, 15 ml of formaldehyde solution was added to each sample. Following a 12 h fixation period, the sperm cells were stained using 10 ml of haematoxylin solution, and the sample was refrigerated at 4°C for 4 days. The sample was then gravity-filtered through a 30 µm nylon mesh to eliminate fine debris, and the stained sperm were gathered via filtration using a 0.6 µm mesh membrane filter (Millipore type GTTP, Merck, Darmstadt, Germany). After air-drying the filter paper containing sperm cells for 1 h, microscope immersion oil was applied to the filter paper to make it transparent before placing it on a glass slide. The sperm on the membrane were counted using an optical microscope (Olympus CX41, Tokyo, Japan) at 400× magnification by examining 50 haphazardly chosen fields (0.1963 mm^2^ per field). Given that the total areas of the membrane filter and the 50 fields were 1150.31 and 9.81 mm^2^, respectively, the total released sperm count was determined from 0.853% of the filter paper’s overall area.

### 2.4. Analysis of mating behaviour

The medaka mating sequence has been described in detail previously [32,33,38,39]. Upon identifying an ovulating female, the male approaches and trails her (‘following’ behaviour). The male then swims quickly around the female (‘quick-circle’ courtship) and wraps her trunk with his dorsal and anal fins (‘wrapping’). The female momentarily trembles to release eggs as the male flexes his body multiple times to release sperm (‘quivering’ behaviour). Once gametes are released, males gradually disengage from females and leave. The female carries the fertilized eggs on her abdomen and, after a few hours, brushes against aquatic plants to attach the eggs to the foliage.

To examine whether these mating behaviours were affected by successive matings, we analysed videos using ELAN v 6.1 annotation software. This analysis allowed the precise measurement of behaviour in terms of (i) mating latency, the time from male introduction into a female tank to the start of male wrapping; and (ii) mating duration, the time from wrapping to leaving [31]. The duration of these periods is probably related to male fatigue over successive matings [38,40]. Further, we analysed (iii) the time spent in following behaviour; and (iv) the number of quick-circle behaviours, both of which are considered key indicators of the intensity of male courtship activity [39,41].

### 2.5. Statistical analysis

The males were allowed to continue mating until they stopped, resulting in 185 matings. Six additional mating experiments were conducted, in which males were allowed to mate the next day. The males and females used in this study were almost identical in terms of standard length and body mass (electronic supplementary material, table S1).

Statistical analyses were conducted using R 4.4.1 (R Core Team, 2024 [42]). In all analyses, linear mixed models (LMMs) or generalized linear mixed models (GLMMs) were implemented using a maximum-likelihood protocol (using lme4) with male ID as a random factor to handle the repeated-measures design. First, all explanatory terms were entered into the full model, and then non-significant terms were sequentially removed from the model by backward elimination using likelihood-ratio tests. The final model included all terms that were significant at *p* < 0.05.

To examine whether sperm depletion occurred during successive matings, we fitted a GLMM with a negative binomial distribution, with the released sperm count per mating as a response variable, the number of matings as an explanatory variable and the number of spawned eggs, male and female body masses, and mating duration as potentially confounding explanatory variables. Three models were generated: one without the use of offset terms and two in which the total number of sperm released in a day by each male (to examine the rate of sperm expenditure per mating) and the number of sperm released during the first mating (to examine the decline rate of sperm expenditure) were used as offset terms.

To examine whether sperm depletion affected fertilization rates, we fitted a GLMM with a binomial distribution, with fertilization rate as a response variable, number of matings as a response variable, number of sperm counted, number of spawned eggs, female and male body masses and mating duration as potential confounding variables.

A GLMM with a negative binomial distribution was used to evaluate the relationship between the number of spawned eggs and the number of matings, the number of sperm counted, male and female body mass or mating duration.

Changes in mating behaviour owing to male fatigue over successive matings were examined. LMMs were used to assess whether mating latency or duration was related to the number of matings, number of sperm counted, number of spawned eggs and male and female body masses. Changes in male courtship behaviour during successive matings were examined. An LMM or a GLMM with a negative binomial distribution was constructed to assess whether the time males spent following females or the number of quick-circle behaviours, respectively, was related to the number of matings, number of sperm counted, number of spawned eggs, male or female body mass and mating duration. The mating latency was added as an offset in the two models.

We differentiated these response variables (including the released sperm count, fertilization rate, number of eggs spawned, mating latency, mating duration, the time males spent following females and the number of quick-circle behaviours) for the first and last mating of the day and next-day mating. Male ID was included as a random factor in the repeated-measures design. The same model settings as those for the GLMM and LMM were used for the analyses. Tukey’s honest significant difference test was used for multiple comparisons.

## 3. Results

### 3.1. Released sperm count during consecutive matings

Individual males mated 19 ± 2 times (mean ± s.d.; range, 4−27; *n* = 10 males; table 1). This variability was significantly related to the time that males spent from their first to their last mating in a day, which ranged from 2.6 to 8.3 h (GLM, $\chi_{2}^{2}$ = 25.68, *p* < 0.0001; electronic supplementary material, table S1). For 7 of the 10 males, we examined their ability to mate the following day. Six succeeded in mating, whereas one failed, despite three females being sequentially presented for spawning (table 1).

**Table 1.** Number of females presented, matings achieved and occurrence of next-day mating by each male medaka (*O. latipes*).

male ID	number of presented females	number of matings achieved by each male	next-day mating
1	25	20	—
2	21	20	—
3	15	13	—
4	33	26	spawned
5	19	16	spawned
6	33	24	spawned
7	17	14	not spawned
8	34	27	spawned
9	5	4	spawned
10	26	21	spawned
mean ± s.d.	22.8 ± 9.3	18.5 ± 2.2	

**Figure 1.**
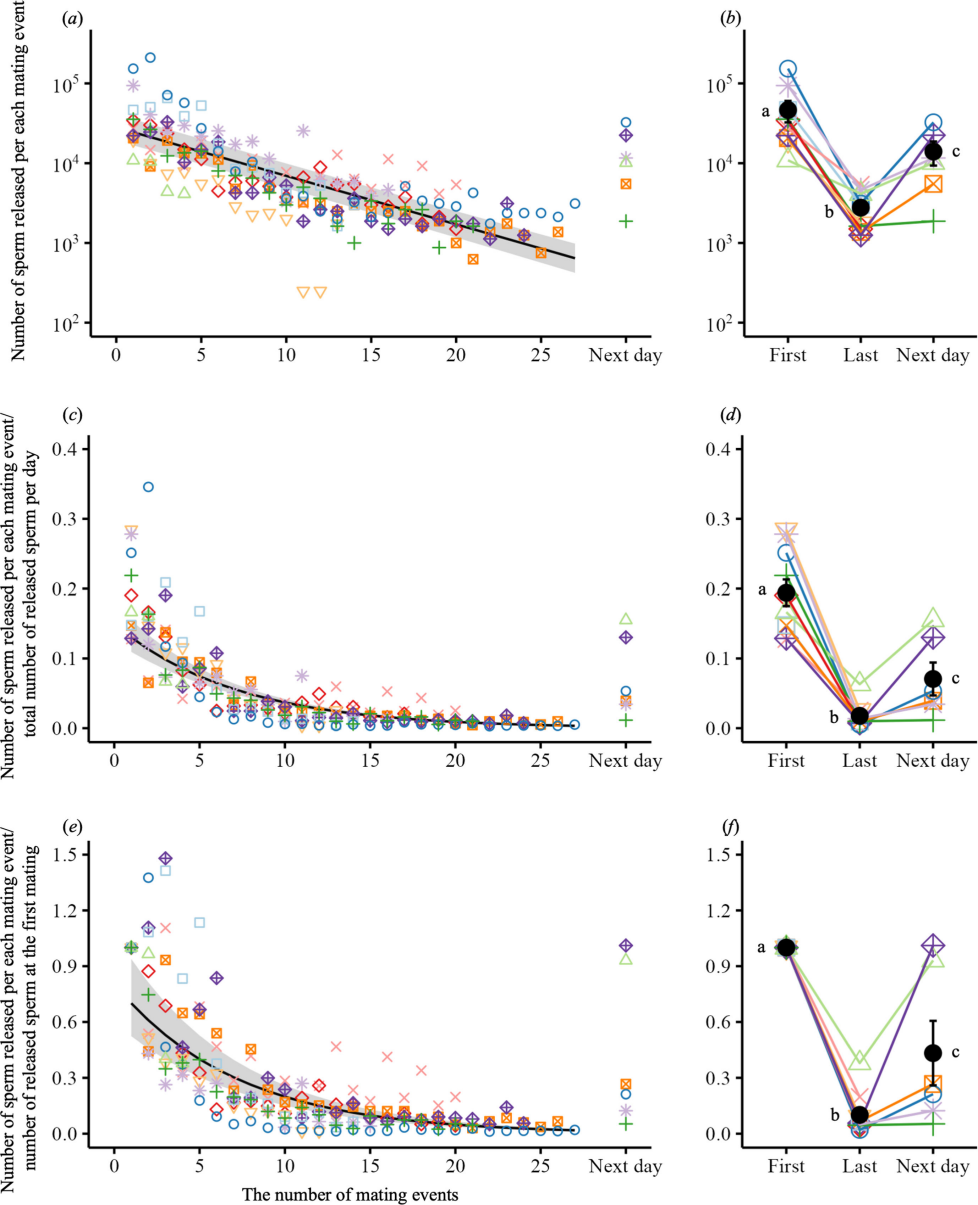
Sperm depletion in male medaka (*O. latipes*) engaging in successive matings. (*a,c,e*) Changes in released sperm count during successive matings. Points with different colours and shapes represent different males. Regression curves are based on GLMMs, with grey-shaded areas indicating 95% confidence intervals. (*b,d,f*) Differences in the released sperm count between the first and last matings on day 1 and during mating the next day. Black points and bars reflect the mean and s.e., respectively. The lowercase letters identify groups that differ significantly, based on Tukey’s post hoc test (*p* < 0.05). (*a,b*) The released sperm count exhibited significant decline ((*a*) negative binomial GLMM, $\chi_{1}^{2}$ = 249.54, *p* < 0.0001; (*b*) $\chi_{2}^{2}$ = 39.71, *p* < 0.0001). (*c,d*) The released sperm count per mating/total released sperm count per day for each male declined significantly with successive matings ((*c*) negative binomial GLMM, $\chi_{1}^{2}$ = 263.21, *p* < 0.0001; (*d*) $\chi_{2}^{2}$ = 35.59, *p* < 0.0001). Total released sperm count per day was entered as the offset in these models. (*e,f*) The released sperm count per mating/the released sperm count at the first mating declined significantly with successive matings ((*e*) negative binomial GLMM, $\chi_{1}^{2}$ = 248.91, *p* < 0.0001; (*f*) $\chi_{2}^{2}$ = 31.97, *p* < 0.0001). The released sperm count at the first mating was entered as the offset in these models.

The released sperm count per mating varied substantially among the consecutive matings, ranging from 250 to 210 875 (mean ± s.d. 12 084 ± 22 365; median, 5125; *n* = 191). The sperm count released during matings 1−3 ranged from tens to hundreds of thousands and declined significantly with successive matings (figure 1*a*; electronic supplementary material, table S2). It decreased from 46 388 ± 44 170 (mean ± s.d.; range, 10 875−153 250, *n* = 10) at the first mating to 2775 ± 1508 (1250−5375, *n* = 10) at the last mating (figure 1*b*). Sperm expenditure at the first mating was 12.8−27.8% of the total sperm that an individual used for a day; this proportion declined significantly during successive matings (figure 1*c*; electronic supplementary material, table S3). During the last mating of the day, it was 0.5−6.3% (figure 1*d*). By matings 4−6, the released sperm count was halved relative to the first mating (figure 1*e*; electronic supplementary material, table S4) and was only 10% at the last mating (figure 1*f*). The released sperm count recovered the following day but was still significantly lower than that at the first mating (figure 1*b*,*d*,*f*). The mean recovery rate was 43.3% (s.d. 42.4%; range, 5.28−101.13%; figure 1*f*).

### 3.2. Fertilization rate and number of spawned eggs

**Figure 2.**
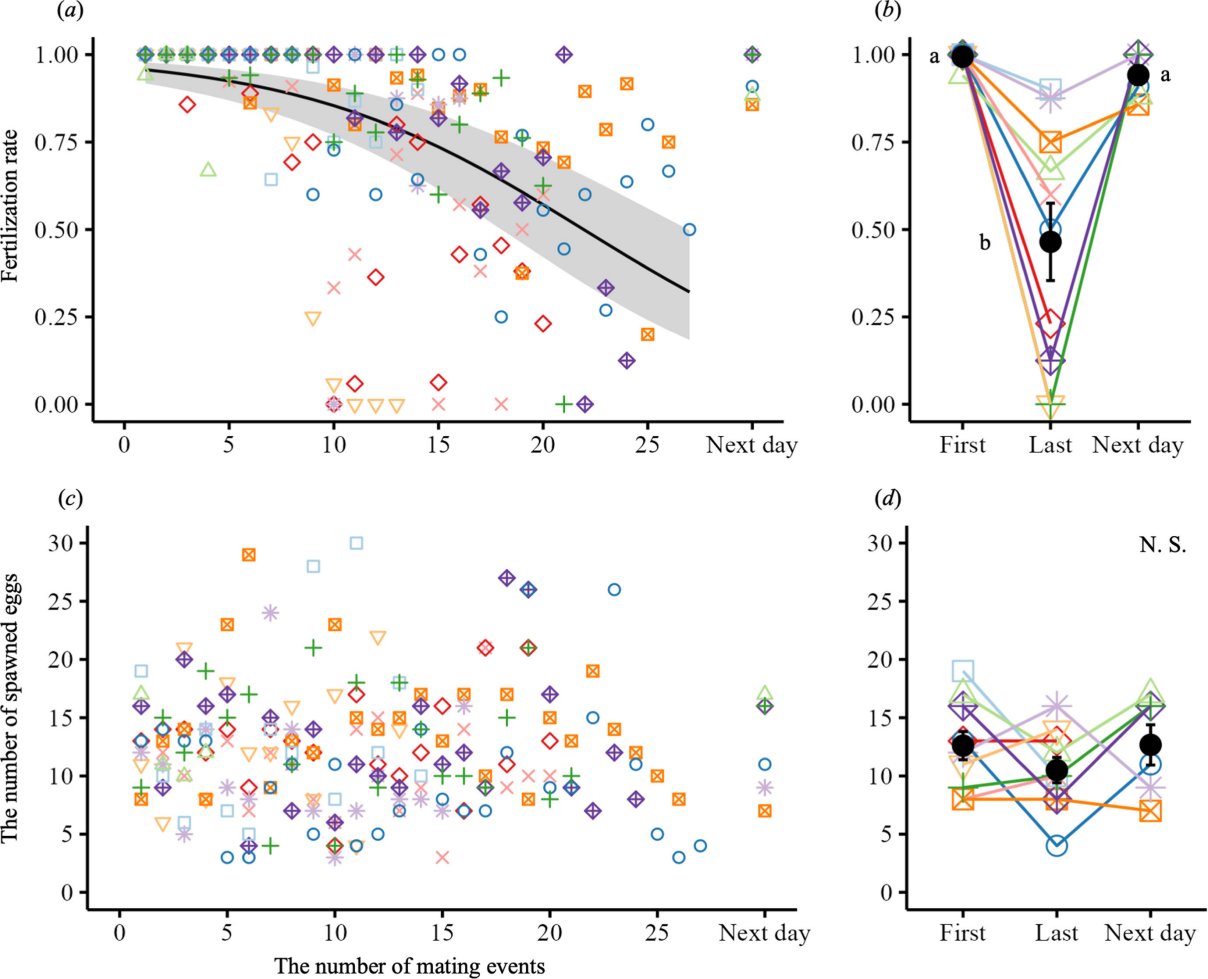
Fertilization rate and number of spawned eggs during successive matings by each male medaka. (*a,c*) Changes in fertilization rate and the number of spawned eggs during successive matings. The points with different colours and shapes represent different males. The regression curve is based on the GLMM, with the grey-shaded area indicating 95% confidence intervals. (*b,d*) Differences in fertilization rate and the number of spawned eggs at the first and last matings of day 1 and during mating the next day. Black points and bars reflect the mean and s.e. respectively. The lowercase letters identify groups that differ significantly, based on Tukey’s post hoc test (*p* < 0.05). (*a,b*) Fertilization rates declined significantly ((*a*) binomial GLMM, $\chi_{1}^{2}$ = 349.42, *p* < 0.0001; (*b*) $\chi_{2}^{2}$ = 124.02, *p* < 0.0001). (*c,d*) The number of eggs spawned did not change during successive matings ((*c*) negative binomial GLMM, $\chi_{1}^{2}$ = 0.004, *p* = 0.95; (*d*) $\chi_{2}^{2}$ = 2.44, *p* = 0.29).

The mean number of eggs spawned was 12 (s.d. 5; range, 3−30; *n* = 185). During the first few matings, the fertilization rate was almost 100%; however, after mating 10, it varied widely from 0% to 100% and declined significantly with successive matings (figure 2*a*; electronic supplementary material, table S5). On the following day, the fertilization rate recovered to 94.1%, with substantial variation (s.d. 6.6, *n* = 6; figure 2*b*), and was significantly higher than that at the last mating (46.5% ± 35.0, *n* = 10) but not significantly different from that at the first mating of the first day (99.4% ± 1.9, *n* = 10). Contrary to our expectations, the number of spawned eggs did not change with successive matings (figure 2*c*,*d*) and was only related to female body mass (electronic supplementary material, table S6). Of the 40 mated females examined, 36 had no residual eggs and four (three after the final mating and one after only one mating) had only one residual egg in their ovaries.

**Figure 3.**
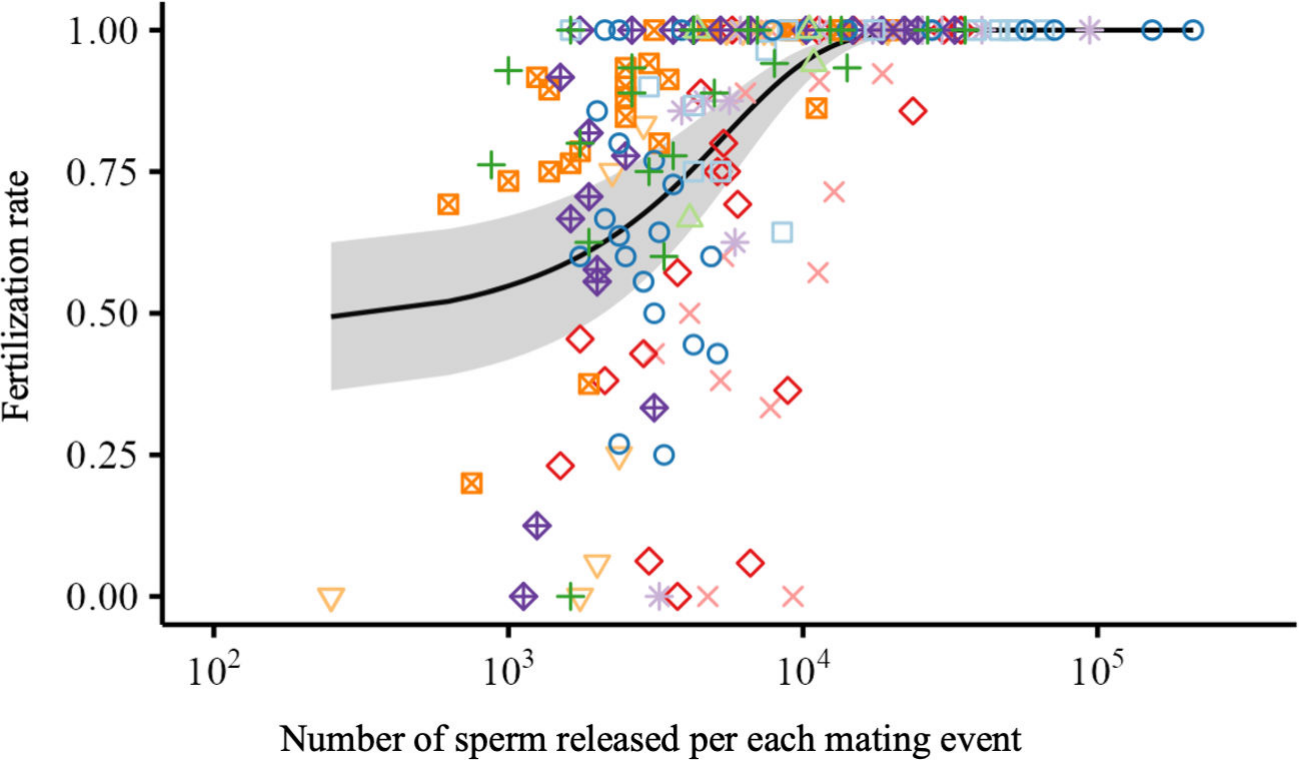
Sperm depletion and fertilization rates in medaka. The points with different colours and shapes represent different males. The regression curve is based on the GLMM, with the grey-shaded area indicating 95% confidence intervals. The fertilization rate was significantly lower when fewer than *ca* 30 000 sperm were released at a mating (binomial GLMM, $\chi_{1}^{2}$ = 21.32, *p*<0.0001).

We examined the relationship between released sperm count and fertilization rate (electronic supplementary material, table S5). When more than *ca* 30 000 sperm were released at each mating, the fertilization rates were almost 100% (figure 3). However, at lower release rates, the fertilization rates were significantly lower because of sperm depletion.

### 3.3. Behaviours related to male fatigue and courtship

**Figure 4.**
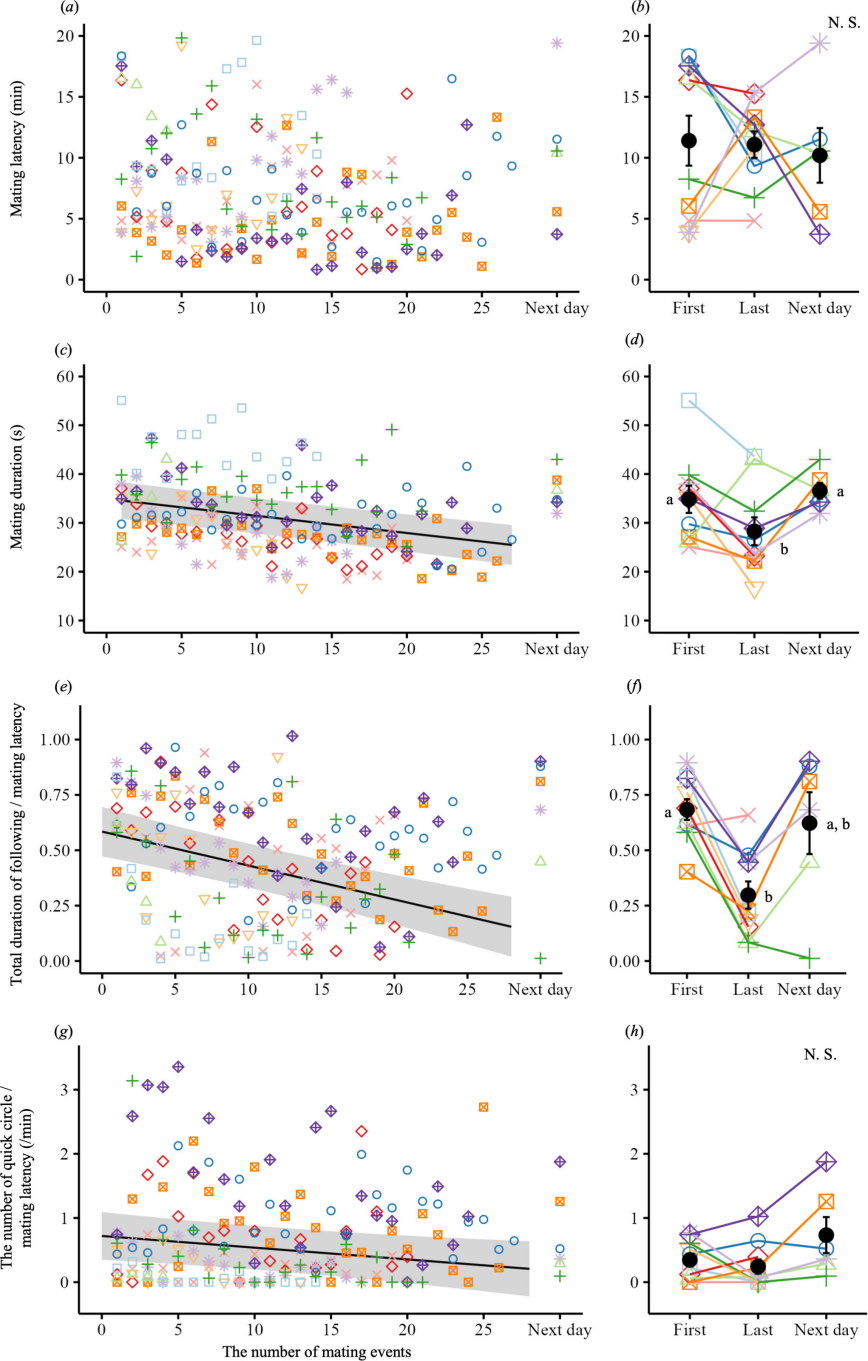
Behaviours related to male fatigue and courtship during successive medaka matings. (*a,c,e,g*) Changes during successive matings in the behaviours examined. The points with different colours and shapes represent different males. The regression lines are based on LMMs, with the grey-shaded areas indicating 95% confidence intervals. (*b,d,f,h*) Differences in the behaviours between the first and last matings on day 1 and during mating the next day. Black points and bars reflect the mean and s.e. respectively. The lowercase letters identify groups that differ significantly, based on Tukey’s post hoc test (*p* < 0.05). (*a,b*) Mating latency (the time from the introduction of a male into a female’s tank to the start of wrapping), did not change during successive matings ((*a*) LMM, $\chi_{1}^{2}$ = 1.42, *p* = 0.23; (*b*) $\chi_{2}^{2}$ = 0.23, *p* = 0.89). (*c,d*) Mating duration (the time from wrapping to leaving), declined significantly ((*c*) LMM, $\chi_{1}^{2}$ = 35.11, *p* < 0.0001; (*d*) $\chi_{2}^{2}$ = 7.63, *p* = 0.0221). (*e,f*) The time males spent following females declined significantly during successive matings ((*e*) LMM, $\chi_{1}^{2}$ = 15.67, *p* < 0.0001; (*f*) $\chi_{2}^{2}$ = 7.14, *p* = 0.028). Mating latency was entered as the offset in these two models. (*g,h*) The number of quick-circle behaviours declined significantly during successive matings ((*g*) negative binomial GLMM, $\chi_{1}^{2}$ = 7.71, *p* = 0.0055; (*h*) $\chi_{2}^{2}$ = 1.70, *p* = 0.43). Mating latency was entered as the offset in these two models.

We examined whether male fatigue changed during consecutive male matings. Overall, mating occurred within 7.1 ± 4.6 min (mean ± s.d.; range, 0.8−19.8 min, *n* = 185) after the introduction of the male into a tank with a new female. The mating latency did not change significantly with consecutive mating events (figure 4*a*,*b*; electronic supplementary material, table S7). The mating duration was 30.9 ± 7.5 s (mean ± s.d.; range, 17−55 s, *n* = 185) and declined significantly with successive matings (figure 4*c*; electronic supplementary material, table S8). The mating duration was significantly shorter at the last mating than at the first and next-day’s matings (figure 4*d*).

We examined whether courtship behaviour changed during consecutive matings. The time that males spent following females decreased with successive matings (figure 4*e*; electronic supplementary material, table S9) and was significantly shorter during the last mating than during the first mating of the day (figure 4*f*). Similarly, the number of quick-circle behaviours declined significantly with successive matings (figure 4*g*; electronic supplementary material, table S10), although it did not differ significantly among the first-, last- and next-day matings (figure 4*h*).

## 4. Discussion

In species capable of multiple matings, lifetime male reproductive success is expected to be determined by the total number of potential mating opportunities [18,21]. Changes in male reproductive investment and mating behaviour owing to consecutive matings can significantly affect the reproductive success of both sexes. In medaka, we explored the dynamics of male reproductive capacity during consecutive matings under unlimited mating conditions. Males mated more frequently than has been previously reported [43,44]. Even under ideal circumstances, males exhibit limited reproductive potential following consecutive mating. We found that males mated 19 times per day on average. Females which mated with males engaging in multiple successive matings experienced reduced fertilization rates. Male courtship behaviours declined with successive matings. Females did not exhibit any counterstrategies such as reduced clutch sizes. These findings imply that sperm is a limited resource for both sexes, potentially leading to sexual conflicts.

Our experiments were not intended to mimic natural conditions, but rather to identify the limits of males’ daily mating capacity and the potential reproductive rate of medaka by experimentally removing factors that limit their reproductive success, such as limited nutrition and mate availability. The observed reduction in sperm release with successive matings, along with incomplete sperm replenishment on the following day, is consistent with prior theoretical and empirical studies demonstrating that males cannot quickly recover from sperm depletion [1,9,45,46]. For example, with the experimental removal of limiting factors, the moth *Ephestia kuehniella* mated approximately five times in its lifetime [19], and the mite *Tetranychus urticae* mated an average of 13 times a day [47]. Therefore, our findings suggest that male medaka do not have an unlimited sperm supply and that the costs associated with sperm production are substantial.

The medaka males exhibited declining counts of released sperm during consecutive matings. On average, males mated 19 times with females, releasing more than 50% of their daily sperm output during the first three matings. Males continued mating despite reaching an average released sperm count of 0.5% of the first count in the last mating of the day. Accordingly, females mating with males undergoing successive mating exhibited reduced fertilization rates, probably because of a reduction in released sperm [36]. This decline in sperm release over the day is consistent with previous findings in various taxa. For example, in fruit flies (*Anastrepha fraterculus*) 45% fewer sperm were released in the second mating than in the first [48], and crabs (*Hapalogaster dentata*) 60% fewer sperm were released in the second mating [17]. In toads (*Bufo bufo*) the released sperm declined by 12% from mating 1 to 3 [49], and in bitterlings (*Rhodeus amarus*) it decreased by 70% from mating 1 to 2 [25]. Given that males are unaware of future mating opportunities, they would enhance their fitness by expending more sperm during earlier mating.

Male courtship effort was lower during later mating. The cost of courtship may discourage males from continuously courting females. Empirical evidence from several taxa has indicated that courtship displays are energetically expensive for males [50–59]. For instance, the moth *Ostrinia furnacalis* beats its wings approximately twice as fast during courtship than during flight, indicating that courtship wing beating incurs high costs [60]. Medaka males incur temporal and energetic costs as they increase their activity in the dark while searching for females to mate with, and persistently follow females during courtship [43,61,62]. To elucidate consecutive mating-associated behavioural changes in males, it may be necessary to assess the temporal and energetic costs of male courtship.

Mating with already-mated males can be costly for females in terms of reduced egg fertilization and offspring production [11,63]. Female medaka are likely to incur this cost because of the marked reduction in released sperm with consecutive mating. Although the fertilization rates declined with successive matings, the males could nonetheless achieve reproductive success, as consecutive matings enabled them to fertilize eggs with fewer sperm. However, when mating with sperm-depleted males, females experience reduced fertilization rates. Therefore, we investigated the possibility that females employ counterstrategies against male sperm depletion. In a previous study on medaka, both fertilization success and courtship frequency decreased as the number of matings increased, indicating that courtship frequency may be an accurate indicator of male quality [37]. Sperm-depleted males continue to mate at the cost of females, although females reduce their clutch size and avoid sperm-depleted males if other males are available [37]. Here, males also exhibited a decline in courtship effort after successive matings, which is consistent with prior findings [37]. However, clutch size did not decline with successive matings. Unlike prior studies, we performed dissections, which revealed that only four of the 40 mated females had only one residual egg, and 36 had no residual eggs in their ovaries. Given that the body size of the females and the number of spawned eggs were smaller in this study than in a previous study [37], larger females capable of spawning more eggs may respond by altering the number of eggs released. For example, female zebrafish allocate more eggs to spawning when mating with larger males [64]. Therefore, female counterstrategies against male sperm depletion warrant further examination.

We discovered that, under ideal conditions, male medaka are capable of mating approximately 20 times per day, whereas females can mate only once per day, quantitatively demonstrating that the potential reproductive rate of males is much greater than that of females. Although medaka serve as a model organism in many fields [65,66], little is known about their ecology in the wild [36]. In the wild, males may face other reproductive constraints including nutritional stress. Consequently, the difference in the potential reproductive rate between males and females is expected to be smaller in the wild than under experimental conditions [67]. To further substantiate our predictions and assess the fitness of our findings, it is essential to investigate the typical mating frequencies and potential reproductive rates of both male and female medaka in the wild.

Our findings revealed changes in the released sperm, number of spawned eggs, fertilization rate and behaviour of medaka males and females during successive matings. Males experienced sperm depletion after successive mating. The rapid decline in released sperm and the reduction in fertilization rates owing to successive matings suggest a cost to females mating with males that have undergone successive matings. Therefore, sexual conflicts exist over limited sperm resources. Future research should investigate male and female mate choice in sperm-depleted males and examine the Coolidge effect [68] (the phenomenon whereby males reduce ejaculate investment in repeated matings with the same female [66]) in medaka with individual identification abilities [69] that possess individual recognition capabilities. Such studies may enhance our understanding of the sexual conflict dynamics surrounding limited sperm resources.

Based on our findings, the cost of male sperm production must be reconsidered to understand sexual selection. We found that even under ideal experimental conditions, where males do not have to search for food or mates or compete with each other, there are limits to their reproductive success with successive matings. In the present study, the male and female reproductive success changed substantially with successive male matings. Therefore, previous studies that examined only the first few mating events may not have provided a comprehensive picture of sexual selection.

## Data Availability

The dataset supporting this article are available at [70]. Supplementary material is available online [71].
